# Clinical characteristics of Chlamydia pneumoniae pneumonia in 145 children: a single-centre retrospective study

**DOI:** 10.3389/fped.2026.1847291

**Published:** 2026-06-25

**Authors:** Yongkang Wang, Tianyu Huang, Weikang Gao, Yun Liu, Wenna Shen, Tingting Yang, Hongchuan Yu, Xinrong Sun

**Affiliations:** Department No. 1 of Respiratory Medicine, Xi'an Children’s Hospital/National Regional Center for Children’s Health, Xi'an, China

**Keywords:** children, *Chlamydia pneumoniae*, clinical analysis, pneumonia, treatment

## Abstract

**Objective:**

This study aimed to summarize the clinical features of *Chlamydia pneumoniae* pneumonia in children.

**Methods:**

We conducted a single-centre retrospective cross-sectional study by retrospectively collecting clinical data from 145 children diagnosed with *Chlamydia pneumoniae* pneumonia at Xi'an Children's Hospital, between January 1 and July 30, 2025. Demographic information, clinical manifestations, laboratory test results, pulmonary imaging findings, and treatment details were analyzed.

**Results:**

Among the 145 children, 89 were male and 56 were female. The median age at onset was 11 years (IQR: 8.33–12.42), and the median disease duration was 14 days (IQR: 12–18). Cough was present in 144 cases (99.3%), with wet cough being predominant (141 cases, 97.2%). Fever was observed in 65 cases (44.8%), primarily low to moderate grade, with a median peak temperature of 38.5°C (IQR: 38.0–39.0). Nasal congestion was noted in 43 cases (29.7%), chest pain in 11 (7.6%), and both rash and hemoptysis in 5 cases each (3.4%). Laboratory results showed a median white blood cell count of 8.09 × 10^9^/L (IQR: 6.43–9.58), a mean neutrophil of 4.65 × 10^9^/L (IQR: 3.44–6.14), a mean lymphocyte of 2.41 × 10^9^/L (IQR: 1.88–2.91), a median neutrophil-lymphocyte ratio of 1.95 (IQR: 1.32–2.99), and a median CRP level of 4.39 mg/L (IQR: 0.86–11.77). Unilateral lung involvement was observed in 109 cases, while bilateral involvement was seen in 36. Bronchoscopy was performed on 36 patients. Regarding treatment, 93 children received azithromycin, and 65 received doxycycline for anti-infective therapy.

**Conclusion:**

Older children who mainly suffer from cough and expectoration, with no fever or low-grade fever, pleural-based lobar consolidation on chest radiography, and normal leukocyte counts and inflammatory biomarkers, are highly likely to be diagnosed with CP pneumonia. Early recognition of these characteristic manifestations can facilitate timely diagnosis and targeted antimicrobial therapy for childhood CPP.

## 1 Introduction

*Chlamydia pneumoniae* (CP) is an atypical pathogen (1) belonging to the *Chlamydiaceae* family. It spreads slowly via respiratory droplets. Due to its lack of a cell wall, it is not susceptible to *β*-lactam antibiotics, making macrolides, tetracyclines, and fluoroquinolones the preferred treatment options. Compared with adult patients with CP infection, children with CP infection frequently present with typical manifestations including persistent cough and low-grade fever and are prone to concurrent infections with other pathogens, which contributes to aggravated illness and prolonged disease course (2). The impact of atypical pathogens such as CP in children is often underestimated due to diagnostic challenges, fluctuating prevalence, and variable disease severity (3). Limited recent research exists on this pathogen, partly because of its absence of clear cyclical epidemic patterns and its nonspecific clinical manifestations. In China, the high incidence of *Mycoplasma pneumoniae*(MP) infection and the widespread empirical use of macrolide antibiotics without pathogen identification may obscure the true prevalence of CP infection. Previous literature has documented that CP infection may lead to a spectrum of respiratory diseases including sinusitis, otitis media, tonsillitis, laryngitis, bronchitis and pneumonia; rare extrapulmonary complications such as erythema nodosum, thyroiditis, encephalitis and Guillain-Barré syndrome have also been sporadically reported (4, 5). The reported incidence of *Chlamydia pneumoniae* pneumonia(CPP) ranges from 3.5% to 16.2% (6–8), though it has been considered relatively uncommon in clinical practice. Children with CP infection mainly present with cough, fever and wheezing, and severe cases may progress to pneumonia (8). Eosinophils(EOS) count levels may serve as a potential biomarker of severe CPP (9). In northern China, CPP exhibits a single epidemic peak, typically occurring between March and July (10). From March to July in northern China, ambient temperature and humidity rise gradually, providing favorable conditions for the survival of CP in nasopharyngeal secretions and aerosols and facilitating pathogen transmission via respiratory droplets and airborne particles. In addition, the resumption of schooling increases close contact risks among school-age children. Meanwhile, spring pollen and cold air irritants in northern regions damage the respiratory mucosal barrier. Collectively, these factors contribute to the observed seasonal epidemic pattern. It predominantly affects the elderly and older children, spreading via droplets. Common clinical presentations include fever, fatigue, headache, sore throat, and cough (11). Radiologically, older children often present with patchy or consolidative opacities, while interstitial changes are more common in neonates (12, 13). Based on a single-center cohort of 145 cases, this study aims to provide a detailed description of the clinical features, laboratory findings, imaging characteristics, and treatment responses of CPP, offering reference for clinical diagnosis and management.

## 2 Methods

This single-centre retrospective cross-sectional study retrospectively collected clinical data from 145 children diagnosed with CPP at Xi'an Children's Hospital, between January 1 and July 30, 2025. Demographic information, clinical manifestations, laboratory tests, pulmonary imaging findings, and treatment details were analyzed. Inclusion criteria included: (1) Clinical manifestations of respiratory infection such as fever, cough, or sputum production, along with pulmonary signs such as moist rales or wheezing, and abnormal lung imaging findings including patchy shadows, consolidation, or air bronchogram signs; (2) Evidence of CP infection via pathogen testing: ① Positive serum CP IgM (titer ≥1:16 considered positive) (4); or ② Positive CP nucleic acid test from throat swab or bronchoalveolar lavage fluid; (3) Age between 1 and 18 years((Infants were excluded because nucleic acid testing cannot differentiate CP from Chlamydia trachomatis, which predominates in children <1 years, particularly <3 months).

Exclusion criteria included: (1) Non-respiratory primary admission diagnosis; (2) Severe underlying diseases (e.g., immunodeficiency, congenital heart disease, neoplastic diseases). (3) Incomplete clinical data.

Limitations of the Diagnostic Approach: The single serum IgM antibody test used in this study is a common screening method for Chlamydia pneumoniae infection in clinical practice. This method has a sensitivity of 85%–90%, but only a specificity of 70%–75% for confirming acute infection. A single positive IgM result without paired sera or documented fourfold rise in IgG titre carries a risk of false-positive results due to serological cross-reactivity with other Chlamydia species and Mycoplasma pneumoniae, which is particularly noteworthy given the high background prevalence of Mycoplasma pneumoniae in the paediatric population. The impact of this diagnostic method on the study results was evaluated through stratified sensitivity analysis by diagnostic approach in subsequent sections.

This study was approved with waiver of informed consent by the Medical Ethics Committee of Xi'an Children's Hospital (Approval No.2026-008-01). The waiver is granted because:(1)The research involves secondary analysis of de-identified retrospective data; (2) No clinical intervention was performed; (3) Privacy protection measures: Remove all patient identifiers (such as names, IDs, addresses, etc.), and the case numbers are generated using an encryption algorithm.

Clinical Data Collection: Clinical data were collected via the hospital's electronic medical record system. This included demographic information, clinical manifestations, physical examination findings, laboratory test results, pulmonary imaging results, bronchoscopy findings, and treatment details.

Nucleic Acid Detection methods: Respiratory pathogen nucleic acid testing was performed on enrolled children via PCR-capillary electrophoresis fragment analysis. The detection panel covered influenza virus A (Flu A), influenza virus B (Flu B), parainfluenza virus (PIV), human bocavirus (HBoV), human rhinovirus (HRV), adenovirus, human coronavirus (HCoV), respiratory syncytial virus (RSV), human metapneumovirus (hMPV), Mycoplasma pneumoniae (MP) and Chlamydia pneumoniae (CP). The commercial kit used was the Multiplex Detection Kit for 13 Respiratory Pathogens manufactured by Ningbo Health Gene Technologies Co., Ltd., and all experimental procedures were strictly implemented in accordance with the manufacturer's instructions.

Bronchoalveolar lavage fluid or blood samples were analyzed using metagenomic next-generation sequencing or targeted next-generation sequencing.

The indications for bronchoscopy include: 1. Pneumonia patients with excessive thick sputum and inability to expectorate; 2.Large area of pulmonary consolidation, persistent unabsorption of pulmonary consolidation or presence of atelectasis. The main purpose is to drain airway secretions and promote lung re-expansion through bronchoscopy.

Imaging Assessment: Two authors independently evaluated lung infiltration based on chest imaging findings and classified it as either bronchopneumonia or lobar pneumonia. Inter-rater reliability for pneumonia classification was assessed using Cohen's kappa coefficient, which yielded a value of 0.827 (95% CI 0.742–0.912), demonstrating excellent agreement between the two independent physicians.

There were no missing values for any variables included in this study.For children with fever, statistics on peak body temperature and duration of fever only included those who exhibited febrile symptoms. Fever was defined as axillary temperature ≥37.3 degrees Celsius. The normality of continuous variables was assessed using the Kolmogorov–Smirnov test. Normally distributed data are expressed as mean ± standard deviation (SD) and were compared using independent samples *t*-tests. Non-normally distributed data are presented as median (IQR) and were analyzed using the Mann–Whitney *U*-test or other nonparametric methods. Categorical variables are described as percentages (%) and were compared using the *χ*^2^ test or Fisher's exact test, as appropriate. Pairwise comparisons were conducted for the variables that showed statistically significant differences in the multi-class outcome, and the correction test level was applied. Data analysis was performed using SPSS 26.0.

## 3 Result

### 3.1 Clinical manifestations

During the study period, 3,011 children with pneumonia were admitted, among whom 145 were diagnosed with CPP, accounting for 4.8% of all pneumonia cases based on etiology. Of the 145 children, 89 were male and 56 were female, with a median age at onset of 11 years (IQR:8.33–12.42). The median disease duration was 14 days (IQR:12–18), and the median hospital stay was 6 days (IQR:5–7). Cough was present in 144 cases (99.3%), of which 141 (97.2%) were productive coughs. Fever was observed in 65 cases (44.8%), mainly of low to moderate grade, with a median peak temperature of 38.5°C (IQR:38.0–39.0). Other symptoms included nasal congestion in 43 cases (29.7%), chest pain in 11 cases (7.6%), and rash and hemoptysis in 5 cases each (3.4%). Pulmonary moist rales were noted in 96 cases (66.2%), and diminished breath sounds in 43 cases (29.7%) (Table 1). Among the patients, 89 (61.4%) showed imaging features consistent with lobar pneumonia. No statistically significant difference in age or gender distribution were found between children with different imaging patterns. Compared to the bronchopneumonia group, the lobar pneumonia group had a higher proportion of children with chest pain (11.2% vs. 1.8%) and diminished breath sounds (44.9% vs. 5.4%, *p* < 0.05) (Table 1). No significant differences were observed in the proportion of fever, peak temperature, duration of fever, disease course, or length of hospital stay between the imaging groups.

**Table 1:** Clinical characteristics of 145 pediatric patients.

Clinical characteristic	Value	Lobar pneumonia	Bronchopneumonia	t/U/X2 value	*p* value
Age (years)	11.00 (8.33–12.42)	11.0 (8.92–12.25)	10.96 (7.96–12.58)	2335.5	0.525
Gender					
Male	89 (61.4%)	58 (65.2%)	31 (55.4%)	1.396	0.237
Female	56 (38.6%)	31 (34.8%)	25 (44.6%)		
Symptoms					
Cough	144 (99.3%)	88 (98.9%)	56 (100.0%)	^a^	1.000
Expectoration	141 (97.2%)	87 (97.8%)	54 (96.4%)	^a^	0.640
Hemoptysis	5 (3.4%)	3 (3.4%)	2 (3.6%)	^a^	1.000
Rhinorrhea	43 (29.7%)	23 (25.8%)	20 (35.7%)	1.606	0.205
Headache	3 (2.1%)	2 (2.2%)	1 (1.8%)	^a^	1.000
Fever	65 (44.8%)	38 (42.7%)	27 (48.2%)	0.423	0.515
Temperature (℃)	38.5 (38.0–39.0)	38.4 (37.9–38.8)	38.6 (38.0–39.0)	421.5	0.222
Fever days	3.0 (1.0–4.0)	3.0 (1.0–5.0)	3.0 (1.0–3.0)	412.5	0.170
Chest pain	11 (7.6%)	10 (11.2%)	1 (1.8%)	4.379	0.036
Rash	5 (3.4%)	4 (4.5%)	1 (1.8%)	^a^	0.649
Gastrointestinal symptoms	15 (10.3%)	8 (9.0%)	7 (12.5%)	0.457	0.499
Crackles	96 (66.2%)	57 (58.9%)	39 (69.6%)	0.481	0.488
Decreased breath sounds	43 (29.7%)	40 (44.9%)	3 (5.4%)	25.821	0.000
Fatigue	3 (2.1%)	3 (3.4%)	0 (0.0%)	^a^	0.284
Laboratory test					
WBC (×10^9^/L)	8.09 (6.43–9.58)	8.12 (6.37–9.49)	7.91 (6.65–10.26)	2372.0	0.626
Neut	4.65 (3.44–6.14)	4.84 (3.55–6.53)	4.58 (3.28–6.95)	2,416	0.436
Lymph	2.41 (1.88–2.91)	2.41 (1.79–2.94)	2.28 (1.76–2.89)	2,611	0.995
NLR	1.95 (1.32–2.99)	1.97 (1.33–2.87)	1.85 (1.22–3.47)	2408.0	0.733
EOS (×10^9^/L)	0.19 (0.12–0.29)	0.20 (0.13–0.31)	0.15 (0.10–0.29)	2078.5	0.093
CRP (mg/L)	4.39 (0.86–11.77)	5.12 (1.56–11.93)	1.76 (0.26–13.00)	1899.0	0.016
PCT (ng/ml)	0.05 (0.03–0.07)	0.05 (0.03–0.07)	0.05 (0.03–0.07)	2361.5	0.578
ALT (U/L)	13 (11–17)	13 (11–17)	13 (10–18)	2452.5	0.872
LDH (U/L)	194 ± 35	194 ± 35	202 ± 36	1.415	0.159
CK-MB (U/L)	13 (12–17)	13 (11–16)	14 (12–19)	2102.0	0.112
Co-detection	27 (18.6%)	17 (19.1%)	10 (17.9%)	0.035	0.851
Pleural effusion	1 (0.7%)	1 (1.1%)	0 (0.0%)	^a^	1.000
Treatment					
Bronchoscopy	36 (24.8%)	34 (38.2%)	2 (3.6%)	22.087	0.000
Pre-admission corticosteroid therapy	13 (9.0%)	8 (9.0%)	5 (8.9%)	0.000	0.990
Pre-admission antibiotic therapy	122 (84.1%)	75 (84.3%)	47 (83.9%)	0.003	0.956
Inpatient corticosteroid therapy	4 (2.8%)	2 (2.2%)	2 (3.6%)	^a^	0.640
Inpatient antibiotic therapy					
Azithromycin	93 (64.1%)	52 (58.4%)	41 (73.2%)	3.268	0.071
Doxycycline	65 (44.8%)	47 (52.8%)	18 (32.1%)	5.936	0.015
Duration days	14.0 (12.0–18.0)	14.0 (12.0–18.0)	15.0(11.0–19.0)	2453.5	0.875
LOS (days)	6.0(5.0–7.0)	6.0(5.0–7.0)	5.9(5.0–6.8)	1990.0	0.059

a Indicates Fisher's exact test was used.

### 3.2 Laboratory findings

The median white blood cell count was 8.09 (IQR: 6.43–9.58) × 10^9^/L, with a mean neutrophil of 4.65 (IQR: 3.44–6.14) × 10^9^/L, a mean lymphocyte of 2.41 (IQR: 1.88–2.91) × 10^9^/L, and a median neutrophil-lymphocyte ratio (NLR) of 1.95 (IQR:1.32–2.99). The median C-reactive protein (CRP) level was 4.39 mg/L (IQR: 0.86–11.77), and the median procalcitonin (PCT) level was 0.05 ng/mL (IQR:0.025–0.07). Pleural effusion was present in only one patient. Children in the lobar pneumonia group had slightly higher CRP levels compared to those in the bronchopneumonia group (5.12 vs. 1.76 mg/L, *p* < 0.05). No statistically significant differences were observed in EOS, PCT, Alanine aminotransferase (ALT), Creatine kinase isoenzymes (CK-MB), or Lactate dehydrogenase (LDH) between the two groups (Table 1).

### 3.3 Etiological detection

Fifty-eight children tested positive only for serum CP-IgM antibodies, 62 children tested positive only for CP nucleic acid via throat swab PCR, and 22 children were positive for both. Two children were positive for multiple pathogens based on targeted sequencing, and one child tested positive for CP via metagenomic next-generation sequencing (mNGS) of bronchoalveolar lavage fluid. Co-detection were identified in 27 children (18.6%). The pathogens included *rhinovirus* (14 cases, 9.7%), *adenovirus* (5 cases, 3.4%), *respiratory syncytial virus* (4 cases, 2.8%), *parainfluenza virus* (2 cases, 1.4%), *Mycoplasma pneumoniae* (3 cases, 2.1%), *bocavirus* (1 case, 0.7%), *Bordetella pertussis* (1 case, 0.7%), and *Moraxella catarrhalis* (1 case, 0.7%). Among school-age children, *rhinovirus* was the most common co-detection(9 cases, 6.2%).

### 3.4 Chest imaging findings

**Figure 1:**
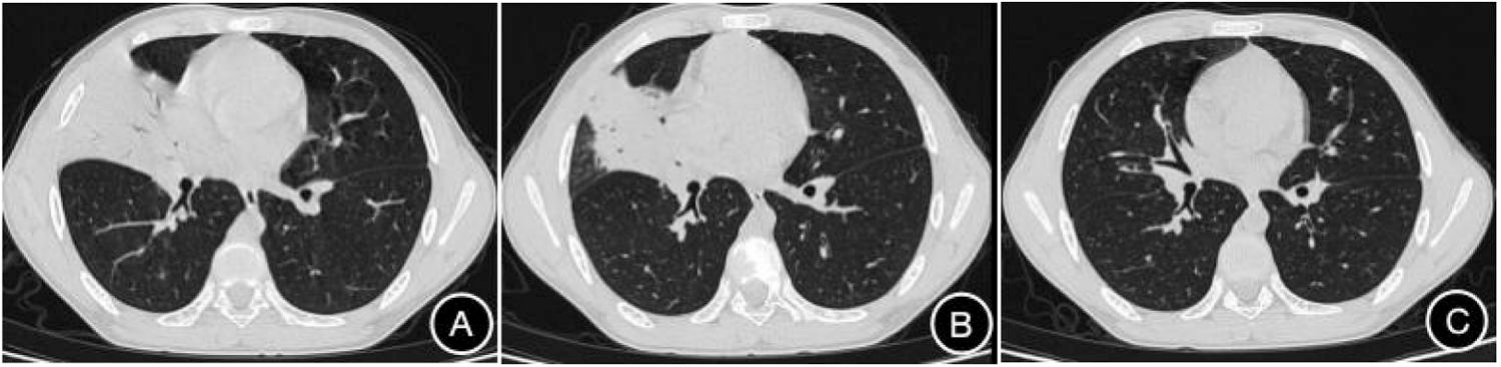
Chest CT imaging of a child with CPP presenting as lobar pneumonia. (12-year-old boy, admitted with cough for 5 days). **(A)** Initial CT shows extensive consolidation in the right middle lobe with relatively clear margins and visible air bronchograms. **(B)** Follow-up CT one week after bronchoscopy shows reduction in the extent of consolidation compared to the initial scan. **(C)** Follow-up CT one month after discharge (39 days after image A) demonstrates near-complete resolution of the consolidation.

**Figure 2:**
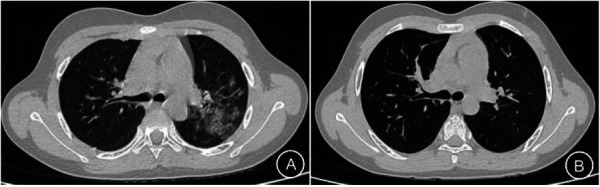
Chest CT imaging of a child with CPP (12-year-old boy, admitted with cough for 9 days and exacerbation accompanied by fever for 4 days). **(A)** Initial CT demonstrates multiple patchy opacities in the left upper lobe with indistinct margins. **(B)** Follow-up CT 13 days later shows significant reduction, fading, and near-complete resolution of the patchy opacities in the left upper lobe compared to the initial scan.

Imaging results showed unilateral lung involvement in 109(75.2%) children. Among these, the left lung was affected in 54(37.2%) cases, with the left upper lobe involved in 16(11.0%) cases and the left lower lobe in 38(26.2%) cases. The right lung was involved in 55(37.9%) cases, with the right lower lobe affected in 21(14.5%) cases, the right middle lobe in 15(10.3%) cases, and the right upper lobe in 19(13.1%) cases. Bilateral lung involvement was observed in 36(24.8%) children (Table 2). A child with CPP presented with a large patchy consolidation shadow beneath the pleura. After treatment, the lesion area began to shrink approximately one week later, and completely absorbed about 39 days later (Figure 1). Another 12-year-old boy presented with a patchy exudative imaging finding, and the lesion was extensive. Just 13 days later, the lesion had largely disappeared (Figure 2).

**Table 2:** Chest imaging findings in 145 children.

Location of imaging findings	*N* = 145
Unilateral	109 (75.2)
Left Upper Lobe	16 (11.0)
Left Lower Lobe	38 (26.2)
Right Upper Lobe	19 (13.1)
Right Middle Lobe	15 (10.3)
Right Lower Lobe	21 (14.5)
Bilateral	36 (24.8)

### 3.5 Bronchoscopy

Bronchoscopy was performed in 36 children (24.8%), of whom 34 belonged to the lobar pneumonia group. Endoscopic examination revealed abundant white flocculent secretions from the lavaged lesion sites in all cases. Six children showed evidence of airway lymphoid follicular hyperplasia, and one child exhibited manifestations of plastic bronchitis. Four children underwent a second bronchoscopic lavage; their chest CT scans all displayed large consolidations adjacent to the pleura.

### 3.6 Treatment

Among the 145 children, 93(64.1%) received azithromycin, with 77(53.1%) treated solely with azithromycin. In 14(9.7%) children, the response to azithromycin was suboptimal, suggesting potential macrolide resistance, and doxycycline was subsequently administered. Glucocorticoids were added in 4 cases. A total of 51(35.2%) children were directly treated with a 10-day course of doxycycline. All children improved and were discharged without adverse outcomes. The proportion of children receiving doxycycline was significantly higher in the lobar pneumonia group compared to the bronchopneumonia group (52.8% vs. 32.1%, *p* < 0.05), whereas no significant differences were observed in the use of azithromycin or glucocorticoids between the two groups (Table 1).

### 3.7 Comparison of the clinical characteristics of the diagnosed children by different diagnostic methods

The children were divided into three groups according to the diagnostic approach: IgM-only positive group (*n* = 58), PCR-confirmed group (*n* = 62), and both positive group (*n* = 22). The results of the multi-group comparison showed that there was a statistically significant difference in the distribution of radiological classification among the three groups (*P* < 0.05). The proportion of lobar pneumonia in the PCR-confirmed group and the both positive group was significantly higher than that in the IgM-only positive group (*P* < 0.05, pairwise *post-hoc* comparison). There were no statistically significant differences in the remaining variables among the three groups, including gender, age, and core clinical symptoms (all *P* > 0.05), as shown in Table 3. The results of pairwise *post-hoc* comparison showed that the proportion of lobar pneumonia in the PCR-confirmed group was significantly higher than that in the IgM-only positive group (*P* = 0.009 < 0.0167). There was no statistically significant difference in the distribution of radiological classification between the PCR-confirmed group and the both positive group (*P* = 0.601 >0.0167), nor between the IgM-only positive group and the both positive group (adjusted *P* = 0.155 >0.0167).

**Table 3:** Comparison of the clinical characteristics of the diagnosed children by different diagnostic methods.

Clinical characteristic	IgM-only positive group	PCR-confirmed group	Both positive group	t/U/X2 value	*p* value
Age	11.00 (8.15–12.69)	10.83 (8.31–12.42)	10.96 (8.77–12.52)	0.215	0.898
Gender				0.752	0.687
Male	33 (56.9%)	40 (64.5%)	13 (59.1%)		
Female	25 (43.1%)	22 (35.5%)	9 (40.9%)		
Symtoms					
Cough	58 (100%)	61 (98.4%)	22 (100%)	^a^	0.720
Expectoration	56 (96.6%)	61 (98.4%)	21 (95.5%)	^a^	0.992
Hemoptysis	3 (5.2%)	1 (1.6%)	1 (4.5%)	^a^	0.638
Rhinorrhea	24 (41.4%)	11 (17.7%)	7 (31.8%)	8.101	0.017
Headache	2 (3.4%)	0 (0)	1 (4.5%)	^a^	0.844
Fever	28 (48.3%)	25 (40.3%)	13 (59.1%)	2.427	0.297
Chest pain	4 (6.9%)	6 (9.7%)	1 (4.5%)	^a^	0.925
Rash	4 (6.9%)	0 (0)	0 (0)	^a^	0.033
Gastrointestinal symptoms	6 (10.3%)	6 (9.7%)	3 (13.6%)	^a^	0.757
Crackles	40 (69%)	43 (69.4%)	10 (45.5%)	4.627	0.099
Fatigue	2 (3.4%)	1 (1.6%)	0 (0)	^a^	0.308
Lobar pneumonia	19(32.8%)	35(56.5%)	11(50%)	6.964	0.031

a Indicates Fisher's exact test was used.

The above results indicated that the baseline characteristics and core clinical symptoms of the cases diagnosed by different methods were highly consistent, with differences only observed in the radiological classification. This difference may be related to the higher diagnostic specificity of PCR testing for acute infection, which can more effectively identify severe cases with more prominent pulmonary lesions.

## 4 Discussion

CP is a recognized cause of community-acquired pneumonia, classified alongside MP and *Legionella* as an atypical pathogen. The reported overall prevalence of CPP varies widely, historically ranging from 3.5% to 16.2% (6–8). In this study, we found a detection rate of 4.8%. The median age was 11 years, with school-age and adolescent children constituting the majority (92.4%). This aligns with a previous study from Beijing (12) but contrasts with several international reports citing younger median ages of 4.7 to 8 years (6, 8, 14). Male children outnumbered females in our cohort, consistent with two prior studies (8, 12), while another report indicated no significant gender difference (15). This may imply that gender is not a strongly associated factor in CPP. The clinical presentation was characterized by a median disease duration of 14 days, with primary symptoms of cough, sputum production, and fever, consistent with previous reports (12, 16). Fever, when present, was predominantly low to moderate grade. Compared to MP pneumonia, the peak fever tended to be lower and the disease course slightly longer. Pulmonary signs were prominent, with moist rales being a common finding. This aligns with a study on neonatal CPP which also reported significant rhonchi and moist rales (13). Diminished breath sounds were significantly more frequent in the lobar pneumonia group. Chest pain was reported in 11 children, all over 6 years of age, and 10 of these had imaging findings consistent with lobar pneumonia. This was likely related to pleural irritation due to subpleural consolidation.

Laboratory markers of extrapulmonary involvement were largely within normal limits in our cohort, suggesting minimal systemic organ damage. This finding is consistent with another Chinese study (13). However, an international study reported a higher burden, with most patients having underlying conditions, 36% experiencing complications, 32% requiring Pediatric Intensive Care Unit (PICU) admission, and one death from myocarditis and cardiogenic shock (8). This stark contrast highlights potential significant regional variations in disease severity. Radiologically, lobar pneumonia was the predominant pattern (61.4% of cases), which is higher than the 40% reported in a Korean study (6). The imaging pattern (lobar vs. bronchopneumonia) showed no correlation with patient age. Unilateral lung involvement was more common, with a predilection for the lower lobes (17).

The diagnosis of CPP relies on etiological testing. A serum IgM titer ≥1:16 and an IgG titer ≥ 1:512 can indicate recent infection. However, IgM antibodies typically appear 2–3 weeks after infection, limiting early diagnosis (16–19). Meanwhile, PCR can detect CP in nasopharyngeal swabs. In this study, 58 children were positive only for serum CP-IgM, 62 were positive only for CP nucleic acid via throat swab PCR, and 22 were positive for both. The sensitivity of PCR for CP is generally lower than that of serology. Therefore, relying solely on PCR or early serological testing alone may underestimate its prevalence (20). Combining serological and molecular detection methods is recommended for accurate pathogen identification (21). CP infection typically does not cause a marked increase in white blood cell count (13, 22), which aligns with findings in our and some previous studies. However, in patients with PCR-positive respiratory samples for CP, 39.3% showed elevated white blood cell counts, and 39.7% had elevated neutrophil percentages (16). Another analysis of pediatric respiratory samples reported leukocytosis in 30% of cases and elevated CRP in 40% (23). Thus, routine laboratory tests alone cannot reliably distinguish CP from viral or bacterial pathogens. For school-age and adolescent children presenting with lobar pneumonia, CP infection should be considered alongside MP*.*

Regarding treatment, macrolides, tetracyclines, and fluoroquinolones are the recommended antibiotics (24). A Korean study reported that 16 out of 21 children were treated with azithromycin (6). In a Chinese study, all 10 children initially received azithromycin, but 5 were later switched to doxycycline, with one ultimately treated with moxifloxacin (12). In our study, 64.1% received azithromycin, but approximately 15% of those showed a suboptimal therapeutic response, necessitating a switch to doxycycline. 51 children received doxycycline as the initial antimicrobial treatment instead of the first-line agent azithromycin, and no child under 6 years of age was treated with doxycycline. This treatment strategy was highly consistent with the current clinical practice and guideline recommendations in the field of pediatric anti-infective therapy.

In recent years, authoritative international academic institutions, including the American Academy of Pediatrics (AAP), have updated their guideline recommendations to explicitly permit the use of doxycycline in children of all ages for short courses (≤21 days), breaking the traditional perception that “tetracyclines are contraindicated in children under 8 years of age” and providing a new treatment option for pediatric pneumonia patients with macrolide resistance. In this study, the core reasons for not using doxycycline in children under 6 years of age were as follows: on the one hand, all children under 6 years of age included in this study had mild pneumonia, and their clinical symptoms and radiological lesions were significantly improved after treatment with first-line agents such as azithromycin, with no indication for doxycycline use; on the other hand, although international guidelines have relaxed the age restriction, in domestic clinical practice, first-line antimicrobial agents with more sufficient safety evidence are still preferred for young children, and doxycycline is only used cautiously in severe children with treatment failure of first-line agents and clear evidence of drug resistance. This treatment logic is fully consistent with the domestic norms for the clinical application of antimicrobial agents in pediatrics.

The results of the 3-group multi-comparison sensitivity analysis stratified by diagnostic approach in this study showed that there were no statistically significant differences in baseline characteristics and core clinical symptoms among the three groups of children, with a significant difference only observed in the radiological classification. The proportion of lobar pneumonia was higher in the PCR-confirmed group. PCR testing has higher diagnostic specificity for acute infection, and can more effectively identify cases with higher pulmonary inflammatory load and more prominent pulmonary lesions. Despite the above differences, the core clinical manifestations of the three groups of children were completely consistent, indicating that the core conclusions of this study would not be affected by essential bias caused by the heterogeneity of diagnostic methods. Meanwhile, the results of this study also suggest that in clinical practice, the diagnosis of Chlamydia pneumoniae pneumonia should combine serological testing and nucleic acid PCR testing to further improve the diagnostic accuracy.

### 4.1 Limitations

First, the single-center design may limit the generalizability. Second, not all patients underwent combined serological and PCR testing, which may have led to an underestimation of the true incidence.Third, we did not include children under 6 years of age with severe pneumonia and treatment failure of first-line agents, so we could not further verify the efficacy and safety of doxycycline in young children with severe disease. Fourth, the lack of post-discharge follow-up data, including serial chest imaging, repeat laboratory testing during recovery, and assessment of longterm outcomes, represents a significant gap. Finally, this study lacks a comparative analysis with pneumonias caused by other pathogens.

## 5 Conclusion

Older children who mainly suffer from cough and expectoration, with no fever or low-grade fever, pleural-based lobar consolidation on chest radiography, and normal leukocyte counts and inflammatory biomarkers, are highly likely to be diagnosed with CP pneumonia. Azithromycin remains the first-line therapy for CPP and yields satisfactory clinical outcomes. For children with poor response or contraindications to azithromycin, doxycycline is a reliable alternative treatment. Early recognition of these characteristic manifestations can facilitate timely diagnosis and targeted antimicrobial therapy for childhood CPP.

## Data Availability

The raw data supporting the conclusions of this article will be made available by the authors, without undue reservation.
